# Factors influencing anti-asthmatic generic drug consumption in Morocco: 1999-2010

**DOI:** 10.1186/2193-1801-3-192

**Published:** 2014-04-16

**Authors:** Imane Ghanname, Samir Ahid, Ghizlane Berrada, Abdelmjid Belaiche, Mohammed Hassar, Yahia Cherrah

**Affiliations:** Research team of Pharmacoepidemiology & Pharmacoeconomics, Faculty of Medicine and Pharmacy of Rabat. Morocco, University Mohammed V -Souissi, Rabat, Morocco; Department of marketing, Pharmed, Casablanca, 20 380 Morocco

**Keywords:** Antiasthmatics, Generic drugs, Originator drugs, Drug consumption, Pricing, Compulsory health insurance, Morocco

## Abstract

**Background:**

The increasing availability of generic drugs (GD) resulted in a remarkable reduction in treatment costs that allowed a better access to health care.The aim of this study is to evaluate the share of anti-asthmatic generic drugs during the period 1999-2010 in Morocco and to look at the factors influencing generic development.

**Methods:**

In this study, we used Moroccan sales data from IMS Health (Intercontinental Marketing Services). The consumption of the drugs was expressed in DDD/1000 inhabitants/day according to the WHO ATC/DDD methodology.

**Results:**

Between 1999 and 2010, anti-asthmatic consumption increased from 3.91 to 14.43 DDD/1000 inhabitants/day. The market of anti-asthmatic generic drugs progressed from 1.83 (47%) to 2.18 (23%) DDD/1000 inhabitants/day from 1999 to 2010. In 2010, inhaled glucocorticosteroids ranked first (0.83 DDD/1000 inhabitants/day), followed by inhaled short acting beta agonists (0.73 DDD/1000 inhabitants/day). The number of brands went from 27 in 1999 to 34 in 2010, with a generic share increasing from 55.55% to 70.59%. The number of anti-asthmatic pharmaceutical preparations increased from 57 to 64 during the same period, of which 31 and 42 were generic preparations. In 2010, the total cost of anti-asthmatic dugs was about 22 million euro, the generics representing 14 million euro.

**Conclusion:**

Despite the introduction of a compulsory insurance scheme called “AMO”, that allows a refund for 69.5% of anti-asthmatic specialties marketed in Morocco, anti-asthmatic generic drug consumption remains limited. The Moroccan market is still largely dominated by the originator drugs with still valid patents.

## Background

According to WHO estimates, there are currently 235 million asthmatic patients in the world (Organisation mondiale de la santé (2012)). Because of its high prevalence, its increasing costs and the possibility to prevent its exacerbations, asthma is a public health priority (Delmas and Fuhrman 2010). Our knowledge of asthma continues to grow rapidly with new concepts and approaches in the management and control of the disease (GINA recommendations) as well as the development of new therapeutic agents (Barnes et al. 1998), which remains the major contributor to the global increase in asthma care spending.

Generic Drugs (GD) are associated with a reduction of costs and according to the WHO recommendations on rational drug use, they are considered as the best practice (Organisation mondiale de la santé (2003)).They are at the heart of economic and social public health policies for facilitating access of drugs to the disadvantaged population and mastering health expenses, not only in poor countries but also in the developed ones; thus the increasing share of generic drug production and consumption (Dussol 2009).

We conducted this study of the Moroccan pharmaceutical market during the period 1999-2010, with the aim of (1) assessing whether the expiration of the Originator Drug (OD) patent and the introduction of its GD was followed by an increase in consumption within the same class of anti-asthmatic drugs, (2) finding the sales share of anti-asthmatic GD and (3) estimating the GD impact on reducing the cost of asthma care.

## Materials and methods

The study uses data from an 11 year period, long enough to eliminate seasonality bias (Abou-Atmé et al. 2006). The data are the number of drugs sold in private pharmacies as reported by the Moroccan subsidiary of IMS Health. They represent 90% of Morocco pharmaceutical consumption. We used the WHO Anatomical Therapeutic Chemical classification system (ATC) (WHO Collaborating Centre for Drug Statistics Methodology 2009). The study involved the main anti-asthmatic classes with the INNs (International Nonproprietary Names) shown in Table 1.

**Table 1:** **Evolution of consumption in DID of the different families of anti**-**asthmatic generic drugs in Morocco**

Class	1999						2010					
	Total Antiasthmatics			Antiasthmatics GD			Total Antiasthmatics			Antiasthmatics GD		
	NS	NP	DID	NS	NP	DID (%)	NS	NP	DID	NS	NP	DID (%)
SABA	7	9	1.95	2	2	0.4 (21.86%)	8	12	3.99	6	8	0.73 (33.48%)
LABA	2	3	0.03	——	——	——	2	2	0.04	1	1	0.0006 (0.03%)
Systemic beta2- agonists	4	14	0.27	3	7	0.15 (8.19%)	6	13	0.35	5	10	0.26 (11.92%)
IC	5	9	0.71	3	4	0.65 (35.52%)	9	13	1.13	7	8	0.83 (38.07%)
LABA-IC	——	——	——	——	——	——	4	12	3.58	2	6	0.18 (8.25%)
Injectable Xanthines	9	22	0.95	7	18	0.63 (34.43%)	5	12	0.38	3	9	0.18 (8.25%)
Total	27	57	3 .91	15	31	1.83 (100%)	34	64	9.47	24	42	2.18 (100%)

*NS*: Number of Specialties / *NP*: Number of Pharmaceutical Presentations.

The consumption data will be expressed in DDDs (Daily Defined Doses) per 1000 inhabitants per day (DID):

The weight of the active ingredient (g) is the product of the number of units per box by the number of boxes sold and the dosage of the active ingredient.

The result was then divided by the DDD assigned by the WHO Oslo Collaborating Center that is “an estimate of the average maintenance dose per day for a drug used for its main indication for a young adult”. A DDD is assigned to each pharmaceutical form of a drug (WHO Collaborating Centre for Drug Statistics Methodology 2009). For active ingredients, as well as drug combinations, not included in the WHO’s ATC classification the daily dose recommended by the VIDAL® dictionary was used as the DDD. To have a daily consumption in DDD/1000 inhabitants/day, we divide the number of obtained DDD by 365 days, and by 1000 inhabitants (Sommet et al. 2005).This unit of measurement allows comparisons at the international level by eliminating the difficulties associated with the heterogeneity of dosage forms, presentations and dosages of drugs across countries.

We used the prices from the IMS health data source to calculate the evolution of the Moroccan Mediun Prices (MMP) of anti-asthmatic in Moroccan dirham (MAD) (1 euro = 11 MAD) to show the impact of generics and new molecules on this mean price.

From a socio-economic aspect of the study, we calculated the Median Monthly Expenditure (*MME*) of treatment of different anti-asthmatic compared to the Guaranteed Minimum Wage (GMW) (High Commission of Planning HCP 2010) in Morocco, to highlight the part of the consumption of these drugs in household spending.

Data were entered and analyzed using SPSS 13.0 software. Quantitative variables were expressed as arithmetic and weighted averages and as standard deviation.

## Results

**Figure 1:**
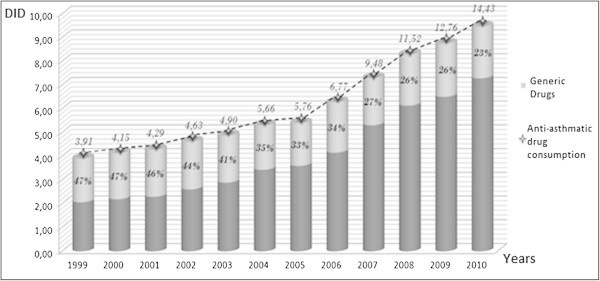
**Evolution of the share consumption in DDD**/**1000 Inhabitants**/**day of anti**-**asthmatic generic drugs in Morocco.**

During our study period from 1999 to 2010, the total consumption of the anti-asthmatic drugs increased from 3.91 to 14.47 DIDs (or an increase of 370%). During this period, the evolution of the share of anti-asthmatic GD can be divided into three phases: a first phase until 2002 where GD represented 47% in 1999 and 41% in 2001, a second phase marked by a decrease in the mid-thirties range (35% in 2002 and 34% in 2006) and a third phase, after 2006, with a marked decline in GD consumption with a share ranging from 27% in 2007 to 23% in 2010 (Figure 1).

Inhaled Corticosteroids (IC) ranked first place in GD consumption (0.65 DID in 1999 and 0.83 in 2010 DID) (Table 1). In this class, the total number of inhaled budesonide DDDs consumed dropped from 0.02 DID (1999) to 0.0005 DID (2010), budenoside does not have a generic presentation. However, beclomethasonehas fully benefited from the introduction of a generic presentation and the number of DID consumed in 2010 were 0.82 DID for the GD versus 0.19 DID for the original drug.

With the introduction of new molecules (from 2 in 1999 to 6 in 2010) and new preparations (2 in 1999 to 8 in 2010) of Short-Acting Beta Adrenergic (SABA), the share of GD in this class has increased from 21.86% in 1999 to 33.48% in 2010 (Table 1). Salbutamol, in the aerosolized form, remains by far the most consumed molecule (1.29 DID for the OD versus 0.40 DID for GD in 1999 and 3.21 DID for the OD versus 0.73 DID for all salbutamol GD in 2010).

Formoterol and salmeterol, Long-Acting Beta Adrenergic drugs (LABA), show a similar evolution (0.01 DID in 1999 versus 0.04 DID in 2010). The introduction of this class GDs had aslight impact on the consumption of the OD in the first two years of its launch to stagnate from 2007 to 0.04 DID, although its share (GD) in sales (€) presented a discount of 97.42% (from €76 920 in 2006 to €1 985 in 2010) (Table 2).

**Table 2:** **Evolution of the sales share of anti**-**asthmatics GD in turnover and percentage consumption of all anti**-**asthmatics drugs**

Class/INN	ATC	1999		2006		2009	
		Sales (€)	%	Sales (€)	%	Sales (€)	%
***SABA***							
Salbutamol	R03AC02	834 612	12.53	1 446 152	19.43	1 631 991	89,73
Terbutaline	R03AC03	——	——	——	——	——	——
Fenoterol	R03AC04	——	——	——	——	——	——
Pirbuterol	R03AC08	——	——	——	——	——	——
Orciprenaline	R03AB03	——	——	——	——	——	——
***Systemic Beta 2 agonists***							
Salbutamol	R03CC02	498 787	45,62	913 079	64,44	1 071 750	68,88
Terbutaline	R03CC03	287 261	100	117 459	100	103 982	100
***LABA***							
Formeterol	R03AC12	——	——	76 920	13.18	1 985	0.33
Salmeterol	R03AC13	——	——	——	——	——	——
***LABA***-***IC***							
Fluticasone + Salmeterol	R03AK06	——	——	——	——	2 038 025	63.36
Budesonide + Formeterol	R03AK07	——	——	——	——	——	——
***IC***							
Beclomethasone	R03BA01	4 077 376	100	7 239 239	94.44	8 908 178	92.62
Budesonide	R03BA02	——	——	——	——	——	——
Fluticasone	R03BA05	——	——	——	——	58 290	5.74
***Injectable Xanthines***							
Theophylline	R03DA04	673 360	59.83	274 693	43.85	204 994	39.81
Diprophylline + Tiemonium iodure	R03DA51	246 457	100	246 385	100	214 387	100
**Total**		6 617 853	71.87%	10 313 927	62.84%	14 233 582	63.50%

——: No GD.

1 € = 11 MAD.

GDs of the injectable xanthenes class were marked by a decline in consumption from 0.63 DID in 1999 to 0.18 DID in 2010 (Table 1). Unlike the only GD of theophylline marketed in Morocco (Xanthium LP®) which showed a marked decrease of 33.50% in volume and 69.60% in value. The generic association diprophylline-timonium (Timozyl®) kept its market share during the study period.

For the LABA-IC fixed combinations, the consumption of the fluticasone-salmeterol has changed substantially. The other association, budesonide-formoterol, that had no generic product, showed a significant increase in consumption (Tables 1 and 2).

Total GD saleswent from €6 million in 1999 to €14 million in 2010, representing 71.87% and 63.50% respectively of the anti-asthmatic market revenues (Table 2). Beclomethasone, the first molecule of the IC class to have a generic on the Moroccan market showed an increase in from €4 million in 1999 to €8 million in 2010, totaling more than half of the anti-asthmatic medications sales. The other half was represented mainly by the SABA class, especially salbutamol. In contrast, the xanthine sales regressed by 54.4% (from €919,817 in 1999 to €419,381 in 2010).

As for the MME, the ratio MME/GMW of anti-asthmatic classes with at least one GD on the Moroccan market decreased gradually between 1999 and 2010, except for the xanthines (Table 3).

**Table 3:** **Evolution of the monthly average cost of anti**-**asthmatic generic drugs compared to guaranteed minimum wage Moroccan asthma**

Class/INN	ATC	1999			2006			2010		
		MME	IQR	MME/GMW	MME	IQR	MME/GMW	MME	IQR	MME/GMW
**SABA**										
Salbutamol	R03AC02	6.02	5.85-6.19	0.04	5.64	5.53-5,81	0.03	5.42	4.38-5.64	0.03
Terbutaline	R03AC03	——	——	——	——	——	——	——	——	——
Fenoterol	R03AC04	——	——	——	——	——	——	——	——	——
Pirbuterol	R03AC08	——	——	——	——	——	——	——	——	——
Orciprenaline	R03AB03	——	——	——	——	——	——	——	——	——
**Systemic Beta 2 agonists**										
Salbutamol	R03CC02	13.33	10.13-22.78	0,09	14.64	10.68-24.58	0.08	12.14	8.05-15.76	0.06
Terbutaline	R03CC03	25.34	15.25-79.45	0,17	12.25	11.48-21.49	0.07	13.05	——	0.07
**LABA**										
Formeterol	R03AC12	——	——	——	5.53	——	0.03	8.53	——	0.04
Salmeterol	R03AC13	——	——	——	——	——	——	——	——	——
**LABA**-**IC**										
Fluticasone + Salmeterol	R03AK06	——	——	——	——	——	——	——	——	——
Budesonide + Formeterol	R03AK07	——	——	——	——	——	——	17.65	6.76-26.73	0.09
**IC**										
Beclomethasone	R03BA01	10.95	8.36-15.30	0,07	5.90	4.02-11.85	0.03	5.90	4.20-12.55	0.03
Budesonide	R03BA02	——	——	——	——	——	——	——	——	——
Fluticasone	R03BA05	——	——	——	——	——	——	17.14	15.76-18.53	0.09
**Injectable Xanthines**										
Theophylline	R03DA04	5.29	4.15-9.88	0,04	3.92	3.47-4.88	0.02	4.19	3.62-4.95	0.02
Diprophylline + Tiemonium iodure	R03DA51	13.42	13.25-14.54	0,09	12.79	12.64-13.90	0.07	12.79	12.64-13.90	0.07

——: No GD.

*MME*: Median Monthly Expenditure; *IQR*: InterQuartile Ranges.

*GMW*: Guaranteed Minimum Wage (€).

MME/GMW: In percentage.

GMW_1999_ = 150.89€.

GMW_2006_ = 182.66€.

GMW_2010_ = 191.74€.

Regarding research ethics, this study is not an experimental research nor carried on humans. It also falls outside of the guidelines of health research ethics in Morocco. The data used for this study is from the Moroccan subsidiary of IMS Health.

## Discussion

Medications are an important component of health care cost. In Morocco, they reached more than 40% of the total health cost in 2007, compared to 18% on average in OECD countries (Commission des Finances et du Développement Economique de la Chambre des Représentants 2009). In Europe, GD prescription shares were around 50% in 2005, with 40% in France, 52% in Germany and 57% in the UK. In United States, the generic market share is more important as it is more than 60% (Zambrowski 2001; Doctisimo 2013; IMS health 2005).

In Morocco, with a volume of 77 million boxes and revenues of €200 million, GDs represented 27% of the pharmaceutical market in 2009. The hospital share of GDs in volume is much higher (80-90%) as the drugs are acquired by public tenders from the lowest bidder. In 2010, GD shares in private pharmacies reached 27.6% in volume and 28.3% in value (Doctisimo 2013; Association Marocaine de l’industrie Pharmaceutique AMIP 2010). Indeed, in 9 years, the use of GD has increased by 6.45 percentage points, from 19.15% in 2001 to 27.6% in 2010, or 0.72% per year (Association Marocaine de l’industrie Pharmaceutique AMIP 2010; Conseil de la concurrence and S.I.S consultant 2009).

In 2009, the respiratory drug class accounted for 45% of the market for the top four pharmaceutical companies among 30 (competition ratios: Cr4 = 45 Cr8 = 72 and Cr20 = 99). Global forecasts estimate that the proportion of GD for respiratory diseases should increase significantly (Chidambaram 2012).

In this study, we analyzed the evolution of the anti-asthmatic GD market shares in volume and value and identified factors influencingits evolution. From 1999 to 2010, the consumption of all classes of anti-asthmatic drugs showed a clear evolution. Indeed, this 11 year period was marked by three major events:

- A high prevalence of respiratory diseases requiring treatment with anti-asthmatic drugs. Asthma and Chronic Obstructive Pulmonary Disease (COPD) are reflecting the high indices of pollution of major and industrialized cities (Rabat, Asthma: 6.6%; Casablanca, Asthma: 12.1% and Chronic Bronchitis: 14%) (Nafti et al. 2009; Chan-Yeung et al. 2004). Pollution represents one of the most important risk factors for asthma (Puddu and Tafforeau 2003), and explains its significant increase in industrialized countries in recent decades, where the prevalence is about 10% (Holgate 1999);

- An evolution of the socioeconomic and socio-cultural level of the Moroccan population with an accentuation of the rural exodus and uncontrolled urbanization (Haut Commissariat au Plan HCP 2010). However, this allowed more access to health care structures and pharmaceuticals products;

- The implementation of a Compulsory Health Insurance (named AMO) in 2006 increased the percentage of the population covered by this scheme (Agence Nationale de l’Assurance Maladie ANAM 2011). Thus patients could afford to buy the expensive new molecules, including the fixed association LABA-IC, as they were reimbursed.

Despite this, the average drug consumption per capita remains low. Encouragement of prescription and consumption of GDs is an important step in allowing the Moroccan population access to affordable drugs. It should be associated with a generalization of the compulsory health insurance and a revision of the Moroccan system of drug pricing (Association Marocaine de l’Industrie Pharmaceutique AMIP 2008). In our study, the evolution of anti-asthmatic GD consumption is not constant and is not the same for all molecules. The majority of IC class consumption, both in volume and in value, is represented by beclomethasone since glucocorticoid therapy is indicated as the first-line treatment of symptomatic asthma (Pujet and Evano-Celli 1995; High Commission of Planning-HCP 2004; Groupe suisse de travail de pneumologie pédiatrique SAPP 2004; Coignard et al. 2001).

Usually, when a molecule loses its patent, expenditures are shifted towards the GDs. It was not the case for salbutamol, which is prescribed, according to GINA, as a treatment for asthma attacks (Global Initiative for Asthma GINA 2011). In 2006, salbutamol represented 50.44% of anti-asthmatic consumption in Morocco (Ghanname et al. 2012). In the United Kingdom it was 58% and in Spain 51%.

The GDs of salbutamol had only a slight evolution during this decade. And the originator product, Ventolin®, ranked 6^th^ of the top-selling drugs in value and 17^th^ in volume (Association Marocaine de l’industrie Pharmaceutique AMIP 2010), representing 10% of GSK’s revenues (Conseil de la concurrence and S.I.S consultant 2009).Its price of €5.11 is aligned with its original price in France (Vidal-Eurekasante 2013) while its cheapest generic, Magistral®, costs €3.64. Despite the its patent expiration in September 1987, Ventolin® sales remained high because the physicians kept prescribing that brand and the patients used it for self-medication.

In Tunisia, a neighboring country with about the same level of development but a better health system on almost all indicators of WHO, Ventolin® costs only €2.27 (-56% of the Moroccan Public Price) (Pharmacie Centrale de Tunisie 2013a). In only three years (2006 to 2008) Moroccan patients paid about 19 million Euro more than Tunisians for Ventolin®, according to the findings of the Finance Committee of the House of Representatives in his informative mission on drug prices in Morocco (Commission des Finances et du Développement Economique de la Chambre des Représentants 2009). This price difference cannot be explained by the size of the market, nor the VAT nor customs duties or by the distribution margins, but by a system of centralized purchasing of imported drugs from all public and private companies via the Central Pharmacy of Tunisia and its bargaining power as recognized by WHO (Conseil de la concurrence and S.I.S consultant 2009). The same system is also adopted by New Zealand that gave it the label of promising model in studies on drug prices worldwide.

The fixed association LABA-IC, fluticasone-salmeterol, marketed by GSK in 2002 as imported SeretideDiskus® has become one of the main drug prescribed because it is more effective (Sbrewsburyss and Britton 2000). This was at the expense of the all classes of anti-asthmatic GDs which went from 46% in 2001 to 33% in 2005.

SeretideDiskus® was protected by another additional few years protection, until September 2013, thanks to “Data Protection” that extends the life of the patent and creates a new barrier to GD access and is considered a “crime” against the pharmaceutical competition (Narendranath 2004; Boulet 2003; Correa 2002; GalaxoSmithKline GSK plc 2002) since it is reduced to the OD only. In 2007, Cooper Pharma introduced Saflu® PMDI with a two-third price reduction, as it is the rule for the first generic. Despite this price difference, the SeretideDiskus® has kept prescribers and patients favors because it is thanks to its easy to use, allowing a good compliance (Khassawneh et al. 2008). In addition, the PMDI requiresthe use of a spacer device in children and the elderly which generates an additional treatment cost.

It was expected that the marketing of the second fixed combination containing budesonide and formeterol (Symbicort Turbuhaler®, AstraZeneca), protected until december 2012, would be accompanied by lower prices due to the competition, but its price was twice the SeretideDiskus® price. The multiplication of devices has introduced other parameters (deposited dose in the bronchi, inspiration force etc.) to prove the effectiveness and determine the price of each specialty (Khassawneh et al. 2008). No generic of this fixed dose combination is yet marketed.

It is a paradox, that in countries with low purchasing power, such as Morocco, the most expensive products are the most sold. The causes lie in the inadequacy of policies to promote the GD and their effectiveness to fight strategies put in place by manufacturers of ODs.

The best indicator of these strategies success or failure is the part of GDs in the volume of sold drugs. The anti-asthmatic GDs have not had the expected success; their share did not exceed 23% in 2010.

In its 2004 study of Moroccan drug prices, WHO, quoted in the report of the parliamentary mission (Commission des Finances et du Développement Economique de la Chambre des Représentants 2009), said that “The predominance of branded drugs in Morocco may be explained by the pricing policy which is biased in favor of the most expensive products”. The manufacturer of the original brand receives, after the expiry of the patent, an additional gain by high profit margins, allowing him to easily invest in advertising campaigns to increase sales of their product and creating a vicious circle at the expense of consumers. A doubt about the quality and efficacy of GDs is created and maintained by the originator manufacturers in the mind of physicians and patients (although officially they say otherwise).

In 2010, a new methodology for pricing the originator drugs was introduced by the Moroccan Ministry Of Health. Itis based on the benchmark of wholesale price, excluding taxes (Gross PPD) in selected countries, including France, Belgium, Spain, Portugal, Greece, Turkey and Saudi Arabia. The set price for any new innovative drug will be the lowest price in these 7 countries. AMIP, the Moroccan pharmaceutical industry association, in its March 2010 document (Association Marocaine de l’industrie Pharmaceutique AMIP 2010), proposes a systematic benchmarking with comparable countries that have managed to get low prices (OD or GD), and whose prices are publicly available, such as Tunisia, Egypt, South Africa and some European countries, and use publicly available databases of essential drugs established and updated regularly by WHO.AMIP proposes also not make a distinction in the pricing procedure between originator drugs whose patent has expired and generic drugs. Their prediction would be an increase of GD consumption from 29% in 2010 to over 50% by 2015.

Despite some efforts, manufacturers of GDs have not yet managed to thwart the strategy of originator drug manufacturers by raising physicians and patients’ confidence in GDs. To overcome their reluctance, among others, a massive communication campaign aimed at the public and the professionals, especially prescribers, should be undertaken. The pharmacist should have the right of substitution while keeping the same benefit margin on the GD as on thecorresponding OD. The health insurance reimbursement policy based on the cheapest generic drug will push originator drug manufacturers to lower their prices as patients become more cost conscious (Zambrowski 2001).

Treatments using anti-asthmatic drugs, like for any other Long Term Disease (LTD), are generally economically burdensome. In fact, they require follow up and some regular treatments for long periods, if not for life. In the absence of health insurance coverage, the situation becomes unbearable for patients, especially the poorest.

France has one of the world’s best health insurance systems. It covers 100% of its population. In Tunisia, 80% of the population is practically covered while in Morocco, two thirds of its citizens remain still excluded from any diseaseinsurance system (Caisse Nationale de Sécurité Sociale CNSS 2011).

In 2006, Morocco launched the compulsory health insurance (AMO, managed by the CNSS and CNOPS) and RAMED schemes. According to the CNOPS data, LTD drug expenditures increased by 40% each year, reaching over €32 million in 2008 (Conseil de la concurrence and S.I.S consultant 2009). They refund 100% of the drug costs for these 41 diseases.

In 2009, more than 2 500 drugs were refundable (about 60% of the drug present in the Moroccan market). It should be recalled that 69.5% among anti-asthmatics specialties are refundable.

The Morocco Competition Council analysis of the cost evolution of generics acquired by the CNOPS showed a glaringdisparity when compared to the originators (Conseil de la concurrence and S.I.S consultant 2009).

In value, GD accounted for only 7% of the CNOPS pharmacy spending. In volume, they represented nearly 30% of drug sales in pharmacies and nearly 90% of the Ministry of Health purchases. Most drugs reimbursed by the CNOPS are called “innovative” (70%) and do not have GDs. But even for those with generics, physicians’ prescriptions are dominated by originator drugs.

The low percentage of GDs purchased by the CNOPS pharmacy can be explained by the scarcity or the absence of GDs for some LTD in one hand, and by the limited use of these GDs when they are available.

To ensure the viability of the AMO, the informative mission on the drug pricing in Morocco suggests that the refund shall be made on the basis of a Reference Price. A drug whose price is higher than the reference price by more than 30% should not be reimbursed. This selective reimbursement will end the uncontrolled proliferation of GDs and exclude immediately from refund all the expensive medications with a generic on the market. If these measures are accompanied by an information campaign targeting prescribers, so that patients are not harmed, they should result in the promotion of GDs and in reductions of more than 40% of the price of these drugs. The mission suggests also that reimbursable drugs by AMO should have a visible distinctive label on their packaging to guide the prescribers and patients.

This decade-long study on the consumption profile of anti-asthmatic GDs in Morocco has some limitations, including the method used to calculate consumption. The DDD is an approximate measurement unit and do not necessarily reflect the daily dose consumed, it does not also take into account the severity of the asthma (Coignard et al. 2001). In addition, the data contain no information on the observance of the treatment, and therefore we cannot assume that the marketed drug had been taken by the patients.

## Conclusion

Contrary to expectations and despite the introduction of the AMO in 2006 and the involvement of the state in promoting GDs, the share anti-asthmatic generics remained limited. This is due to several factors: ones inherent to the generic drug situation in Morocco and the others specific to this class of therapeutics - sociocultural context, traditional healing, difficulty using the device, patients not aware of the gravity of their disease.

## Abbreviations

WHO: World Health Organization
GINA: Global INitiative for Asthma
GD: Generic Drug
OD: Originator Drug
ATC: Anatomical Therapeutic Chemical
INN: International Nonproprietary Name
DDD: Daily Defined Dose
DID: DDD/1000 Inhabitants/Day
MAD: Moroccan Dirham
MME: Median Monthly Expenditure
GMW: Guaranteed Minimum Wage
IC: Inhaled Corticosteroids
SABA: Short Acting Beta Agonist
LABA: Long Acting Beta Agonist
AMO: Compulsory Health Insurance
PMDI: Press. Meter Dose Inhaler
AMIP: Moroccan Association of Pharmaceutical Industry
LTD: Long-term disease
CNSS: National Social Security Fund (Private Employees)
CNOPS: National Social Welfare Fund (Civil Servants)
RAMED: Medical Assistance Regimen (Indigents, Needy).
